# Heparanase and macrophage interplay in the onset of liver fibrosis

**DOI:** 10.1038/s41598-017-14946-0

**Published:** 2017-11-02

**Authors:** Maria Francesca Secchi, Marika Crescenzi, Valentina Masola, Francesco Paolo Russo, Annarosa Floreani, Maurizio Onisto

**Affiliations:** 1 University of Padova, Dept. Biomedical Sciences, 35121 Padova, Italy; 2 University of Padova, Dept. of Surgery, Oncology and Gastroenterology, 35124 Padova, Italy; 3 University of Verona, Dept. of Medicine, 37134 Verona, Italy

## Abstract

The heparan sulfate endoglycosidase heparanase (HPSE) is involved in tumor growth, chronic inflammation and fibrosis. Since a role for HPSE in chronic liver disease has not been demonstrated to date, the current study was aimed at investigating the involvement of HPSE in the pathogenesis of chronic liver injury. Herein, we revealed that HPSE expression increased in mouse livers after carbon tetrachloride (CCl_4_)-mediated chronic induction of fibrosis, but with a trend to decline during progression of the disease. In mouse fibrotic liver tissues HPSE immunostaining was restricted in necro-inflammatory areas, co-localizing with F4/80 macrophage marker and TNF-α. TNF-α treatment induced HPSE expression as well as HPSE secretion in U937 macrophages. Moreover, macrophage-secreted HPSE regulated the expression of α-SMA and fibronectin in hepatic stellate LX-2 cells. Finally, HPSE activity increased in the plasma of patients with liver fibrosis but it inversely correlated with liver stiffness. Our results suggest the involvement of HPSE in early phases of reaction to liver damage and inflammatory macrophages as an important source of HPSE. HPSE seems to play a key role in the macrophage-mediated activation of hepatic stellate cells (HSCs), thus suggesting that HPSE targeting could be a new therapeutic option in the treatment of liver fibrosis.

## Introduction

Chronic liver injury is characterized by necrosis and inflammation, which trigger fibrosis and may eventually lead to cirrhosis and organ functional failure. Activated Kupffer cells and hepatic stellate cells (HSCs) are the main cell types involved in the pathogenesis of liver fibrosis. Kupffer cells promote inflammatory and fibrogenic responses by releasing cytokines, chemokines and growth factors that exacerbate inflammation and trigger the activation of HSCs, a central event during liver fibrosis. Quiescent HSCs are activated into fibrogenic α-SMA positive myofibroblasts by a plethora of paracrine and autocrine stimuli and they profoundly alter the microenvironment by the secretion of excessive extracellular matrix (ECM) proteins (collagens, fibronectin, laminin). All these events aim at recovering and maintaining organ functions but, when deregulated by prolonged injury, result in uncontrolled fibrogenesis, ECM accumulation and disrupted organ architecture^1–4^.

In mammals, among the enzymes that degrade ECM and basal membranes, heparanase (HPSE) is the only one with the ability to degrade heparan sulfate (HS) chains of heparan sulfate proteoglycans (HSPGs), generating fragments of about 5–7 kDa. At intracellular level (endosomal and lysosomal), HPSE participates in the turnover of membrane-associated HSPGs, while the secreted enzyme is involved in the remodeling and degradation of ECM^5,6^. In physiological conditions, HPSE expression is tightly regulated to prevent uncontrolled HS cleavage and adverse biological effects. Conversely, HPSE expression is up-regulated in several pathologies^7–9^. It is well accepted that HPSE enzymatic activity contributes to glomerular basement membrane disassembly and proteinuria in several experimental and human glomerulopathies^10–13^ as well as to sustain angiogenesis and tumor cell migration in cancer progression^14–16^. Interestingly, recent findings on inflammatory disorders of the intestinal tract have also pointed to HPSE as an important link between inflammation and cancer^17,18^. Beyond matrix remodelling, extracellular HPSE activity affects both physiological and pathological processes if considering the broad range of molecules (growth factors, cytokines, chemokines and enzymes) that negatively charged HS bind and that are left to diffuse upon HPSE cleavage^15,19,20^. Increased HPSE activity at renal tubular level was demonstrated to regulate epithelial-to-mesenchymal transition (EMT) of proximal tubular cells, creating a pro-fibrotic milieu. Indeed, we have recently shown that, by regulating the availability and activity of growth factors (i.e. FGF-2 and TGF-β), HPSE promotes tubular EMT and kidney fibrosis^21,22^. Consistently, in the streptozotocin-induced diabetic nephropathy model, HPSE-KO mice showed less interstitial fibrosis compared to untreated mice^23^.

Although the role of HPSE as a pro-cancerous agent has been widely characterized in hepatocarcinoma (HCC)^24–26^, its involvement in non-cancerous chronic liver disease is poorly understood thus far and controversial results have been obtained both from human tissues and animal models of liver fibrosis. The comparison performed by Xiao *et al*. on HPSE expression in normal, cirrhotic and cancer livers did not reveal significant differences in mRNA and protein levels between normal and cirrhotic tissues compared with the increased levels in HCC tissue^24^. Surprisingly, Ikeguchi *et al*. found a decreased amount of HPSE mRNA in HCC tumors compared to adjacent non-cancerous tissue. Moreover, HPSE expression in non-cancerous tissue was found to negatively correlate with fibrosis stage^27^. Two different experimental studies demonstrated increased HPSE protein levels in the fibrotic liver of thioacetamide-treated rats although discordant results regarding the fibrotic stage of the up-regulation emerged and no deep investigation in HPSE regulation and possible effects were performed^28,29^.

In the current study, to shed light on HPSE involvement in liver fibrosis, the tempo-spatial pattern of HPSE expression in the well-established animal model of CCl_4_-induced fibrosis was investigated. The evidence from *in vivo* data, demonstrating increased HPSE expression in the early phases of chronic disease, prompted us to examine the cellular mechanism involved in such a condition. A role for inflammatory macrophages in mediating HPSE accretion was demonstrated as well as HPSE effects on HSCs activation were addressed. To translate our observation, HPSE activity was also investigated in the plasma of patients with chronic liver diseases at different stages.

## Results

### HPSE was up-regulated in mice with early chronic liver injury

**Figure 1:**
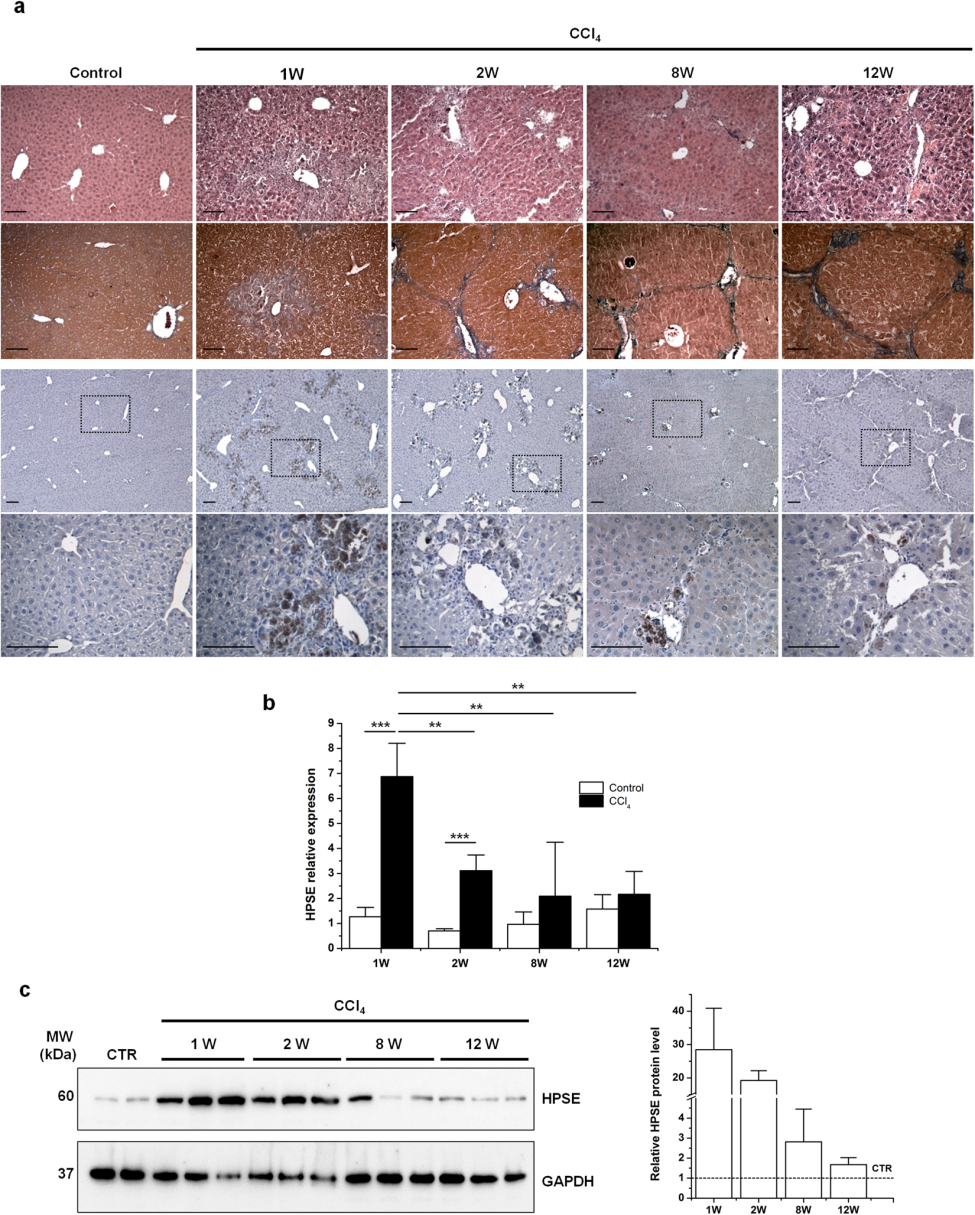
Liver fibrosis and expression of HPSE in mice with CCl_4_-induced chronic liver disease. (**a**) Histological assessment of CCl_4_-induced liver fibrosis through H&E staining (upper panels) and Azan-Mallory staining (medium panels) of control and CCl_4_-injured livers at the indicated time of treatment. Lower panels show immunohistochemistry for HPSE on liver sections from control and CCl_4_-treated mice at the indicated time points. Scale bars = 100 μm. **(b)** HPSE mRNA expression in livers from control and CCl_4_-treated mice for the indicated time, measured by real-time RT-PCR analysis and normalized to GAPDH. Error bars represent s.e.m., *n* = 3, ***p < 0.001, **p < 0.01. **(c)** Western blot analysis for HPSE on whole liver protein extracts from CCl_4_-induced fibrotic mice and control mice. Coomassie blue staining was used as a loading control (Supplementary Fig. S5). Full-length blots are presented in Supplementary Fig. S6.

The tempo-spatial expression of HPSE in liver fibrosis was studied in mice chronically intoxicated with CCl_4_, which represent a well establish model of fibrosis induction. To evaluate the outcome of the treatment in terms of histopathological changes and hepatic fibrosis, H&E and Azan-Mallory stainings were respectively performed on control and CCl_4_-treated livers. Normal lobular architecture and strictly vascular distribution of collagen fibers were observed in liver sections from control mice at each point in time. Periodic administration of CCl_4_ in mice caused massive centrilobular hepatic inflammation and necrosis accompanied by perisinusoidal fibrosis in early chronic injured livers. Liver tissues from 8 and 12 week CCl_4_-treated mice showed mild cell infiltration, pseudolobules formation and bridging fibrosis (Fig. 1a, upper panels). Immunostaining for α-SMA marked vascular smooth muscle cells in control livers while activated HSCs at the site of injury and in correspondence to fibrotic septa were detected after CCl_4_ treatment (Supplementary Fig. S1). After 1 and 2 weeks of CCl_4_ exposure, the expression of hepatic HPSE was found increased, as determined by immunohistochemistry whereas a weak positivity was observed after 8 and 12 weeks. In addition, immunostaining for HPSE revealed a centrilobular localization, nearby necrotic areas with perisinusoidal fibrosis and inflammatory cell infiltrate (Fig. 1a, lower panels). As shown in Fig. 1b, hepatic HPSE mRNA levels were significantly up-regulated in the liver of mice with early chronic disease but not in mice with advance disease, compared with control animals. Moreover, HPSE mRNA levels were significantly reduced in livers of 2 week- compared to 1 week-treated group. In line with real-time RT-PCR data, HPSE protein level reached a peak at 1 and 2 weeks of CCl_4_ injection while declining at 8 and 12 weeks of treatment (Fig. 1c).

### HPSE co-localized with macrophages in early chronic CCl_4_-injured livers

**Figure 2:**
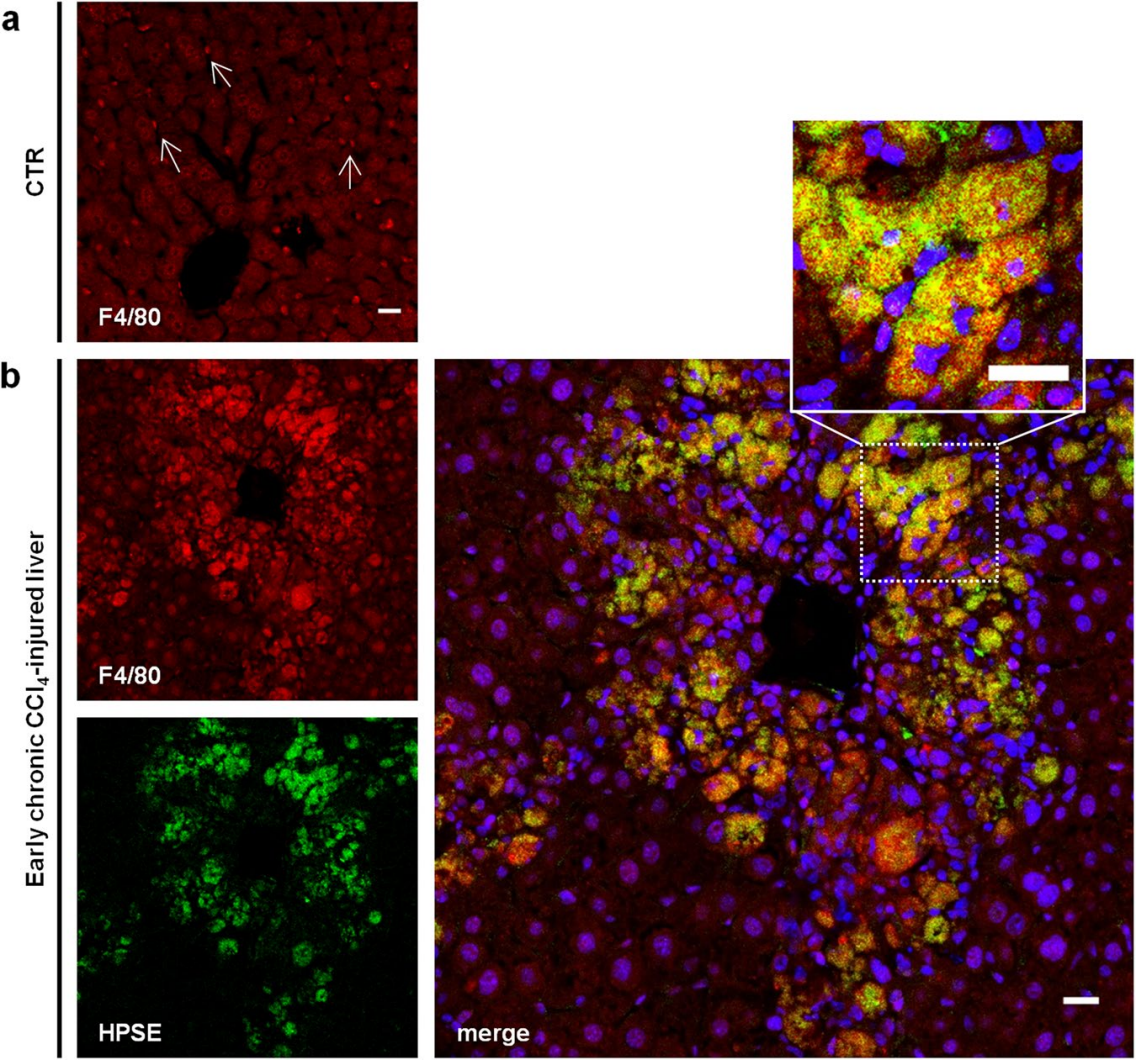
HPSE co-localization with F4/80 in early CCl_4_-injured liver tissues. **(a)** Confocal immunofluorescence staining for F4/80 on control livers showing Kupffer cells lining sinusoid. White arrows indicate representative cells. **(b)** Representative image of confocal double immunofluorescence staining for HPSE (green) and F4/80 (red) on early chronic CCl_4_-injured livers (1 week of CCl_4_ administration). Cell nuclei were counterstained with Hoechst (blue). Insert shows high magnification from the white box. Scale bars = 20 μm.

Starting from the observation that HPSE was localized mainly around the necro-inflammatory areas, we speculated that liver macrophages could be a putative cell source of HPSE in toxic injured liver. To investigate whether macrophages could be involved in the HPSE up-regulation, a double immunofluorescence staining for HPSE and F4/80 (a mouse macrophage-specific marker) was performed in early chronic CCl_4_-injured livers. In healthy mouse livers, F4/80 decorated resident Kupffer cells lining hepatic sinusoids (Fig. 2a). In the livers of mice treated for 1 and 2 weeks with CCl_4_, we detected enhanced F4/80 immunoreactivity indicating an increased macrophages infiltration. HPSE immune-labelling strongly co-localized with F4/80 after 1 and 2 weeks of CCl_4_ administration, consistent with our hypothesis that macrophages are a relevant source of HPSE in CCl_4_-injured liver (Fig. 2b).

### Immune regulation of HPSE expression in macrophages

**Figure 3:**
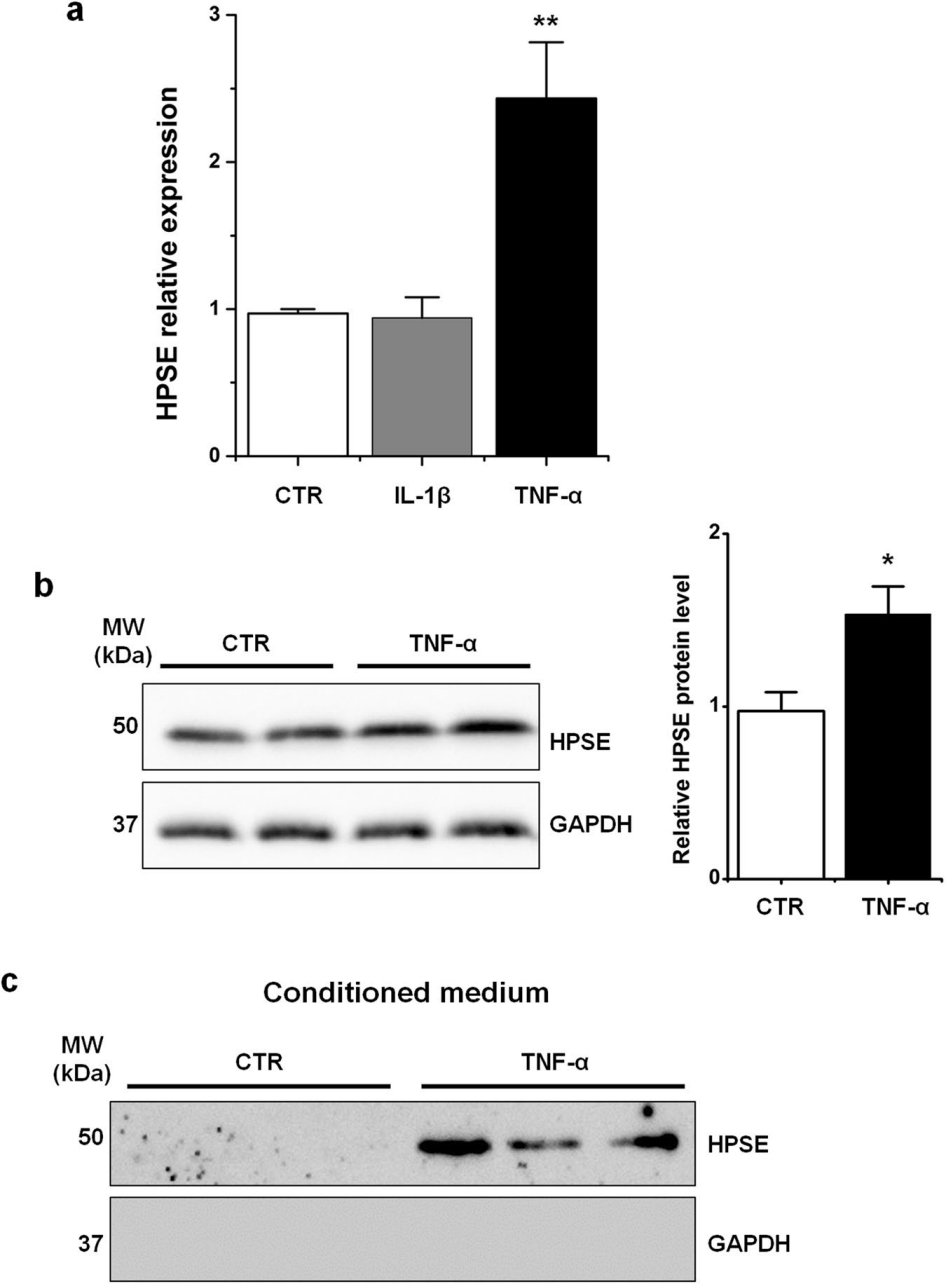
TNF-α regulation of HPSE expression and secretion in U937 macrophages. (**a)** U937 cells were treated with IL-1β and TNF-α for 24 h. The expression of HPSE was assessed by real-time RT-PCR and normalized to GAPDH. Error bars represent s.e.m., *n* = 3, **p < 0.01. **(b)** Western blot analysis for HPSE on lysates from control and TNF-α-treated U937 cells. Error bars represent s.e.m., *n* = 3, *p < 0.05. Full-length blots are presented in Supplementary Fig. S7. **(c)** Western blot analysis for HPSE on conditioned medium from control and TNF-α-treated U937 cells. Full-length blot is presented in Supplementary Fig. S8.

**Figure 4:**
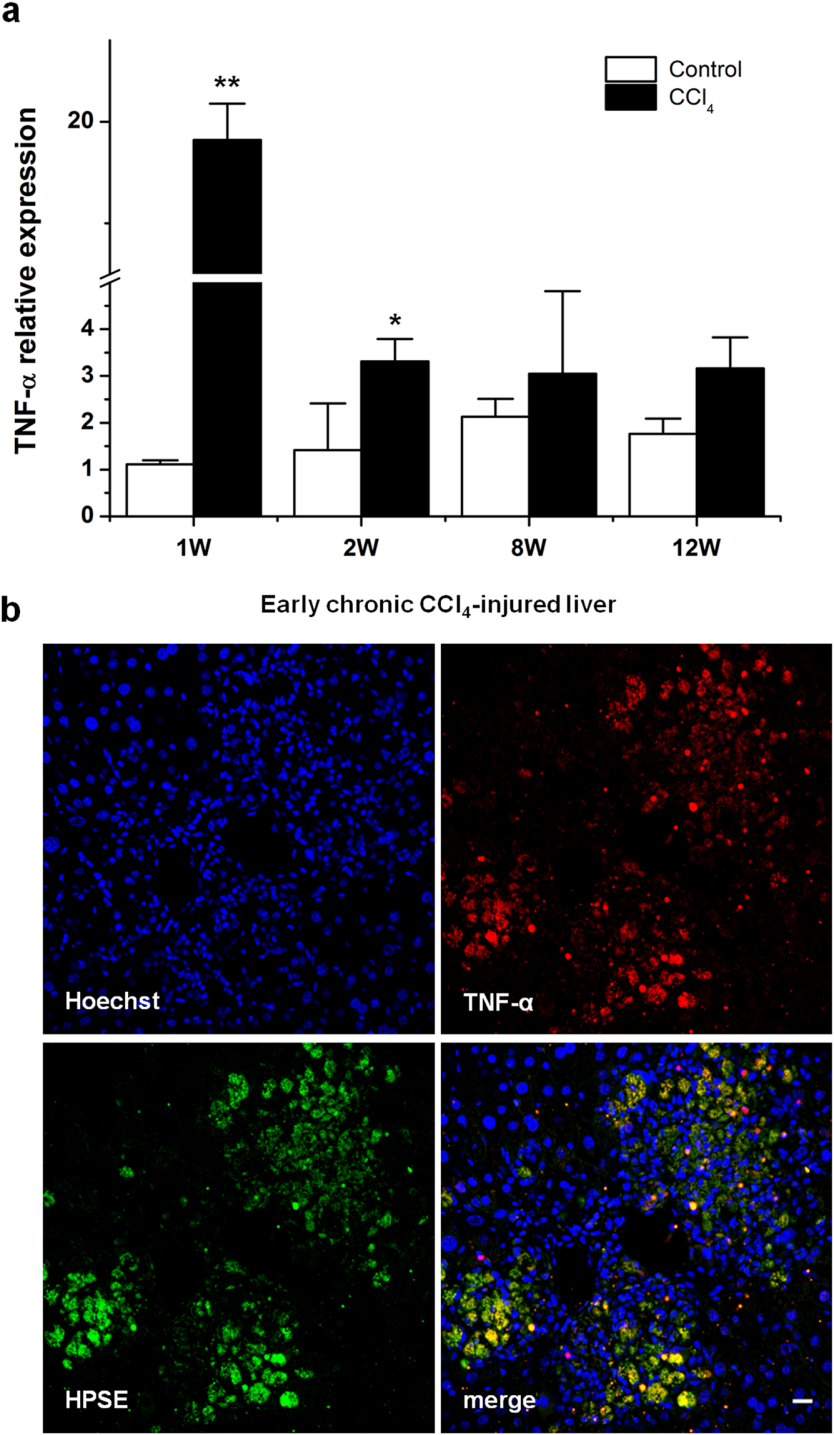
HPSE co-localization with TNF-α in early CCl_4_-injured liver tissues. (**a)** TNF-α mRNA expression in livers from control and CCl_4_-treated mice, measured by real-time RT-PCR and normalized to GAPDH. Error bars represent s.e.m., *n* = 3, **p < 0.01, *p < 0.05. **(b)** Representative image of confocal double immunofluorescence staining for TNF-α (red) and HPSE (green) on early chronic CCl_4_-injured livers (1 week of CCl_4_ administration). Cell nuclei were counterstained with Hoechst (blue). Scale bar = 20 μm.

To gain insight into the mechanism of HPSE immune regulation upon liver injury TNF-α and IL-1β, the main pro-inflammatory mediators in the initial phase of hepatic injury, and TGF-β, as an anti-inflammatory cytokine, were tested on U937 macrophages to measure HPSE expression. TNF-α treatment induced a significant up-regulation of HPSE expression, both at mRNA and protein levels (Fig. 3a,b). We also proved that HPSE protein was undetectable in the conditioned medium of U937 cells but it was released in response to TNF-α stimulation (Fig. 3c). Differently from TNF-α, IL-1β did not affect HPSE mRNA levels (Fig. 3a). Data on IL-1β were also confirmed in RAW 264.7 cell line (data not shown). U937 treated with TGF-β significantly increased HPSE mRNA levels (Supplementary Figure S2a). Moreover, TGF-β induced the secretion of HPSE in the medium of U937 (Supplementary Figure S2b). To further confirm our observations, TNF-α and TGF-β mRNA expression were measured in the fibrotic livers of mice treated with CCl_4_. Compared to control mice, in CCl_4_-injured livers TNF-α mRNA levels were up-regulated at 1 week and at 2 weeks. On the contrary, no statistically significant difference was found after 8 weeks and 12 weeks of CCl_4_ treatment (Fig. 4a). Similar results were observed for TGF-β (Supplementary Figure S3). Moreover, HPSE co-localized with TNF-α in fibrotic mouse livers after 1 and 2 weeks of CCl_4_ administration (Fig. 4b).

### Inhibition of HPSE attenuated macrophage-mediated HSCs activation

**Figure 5:**
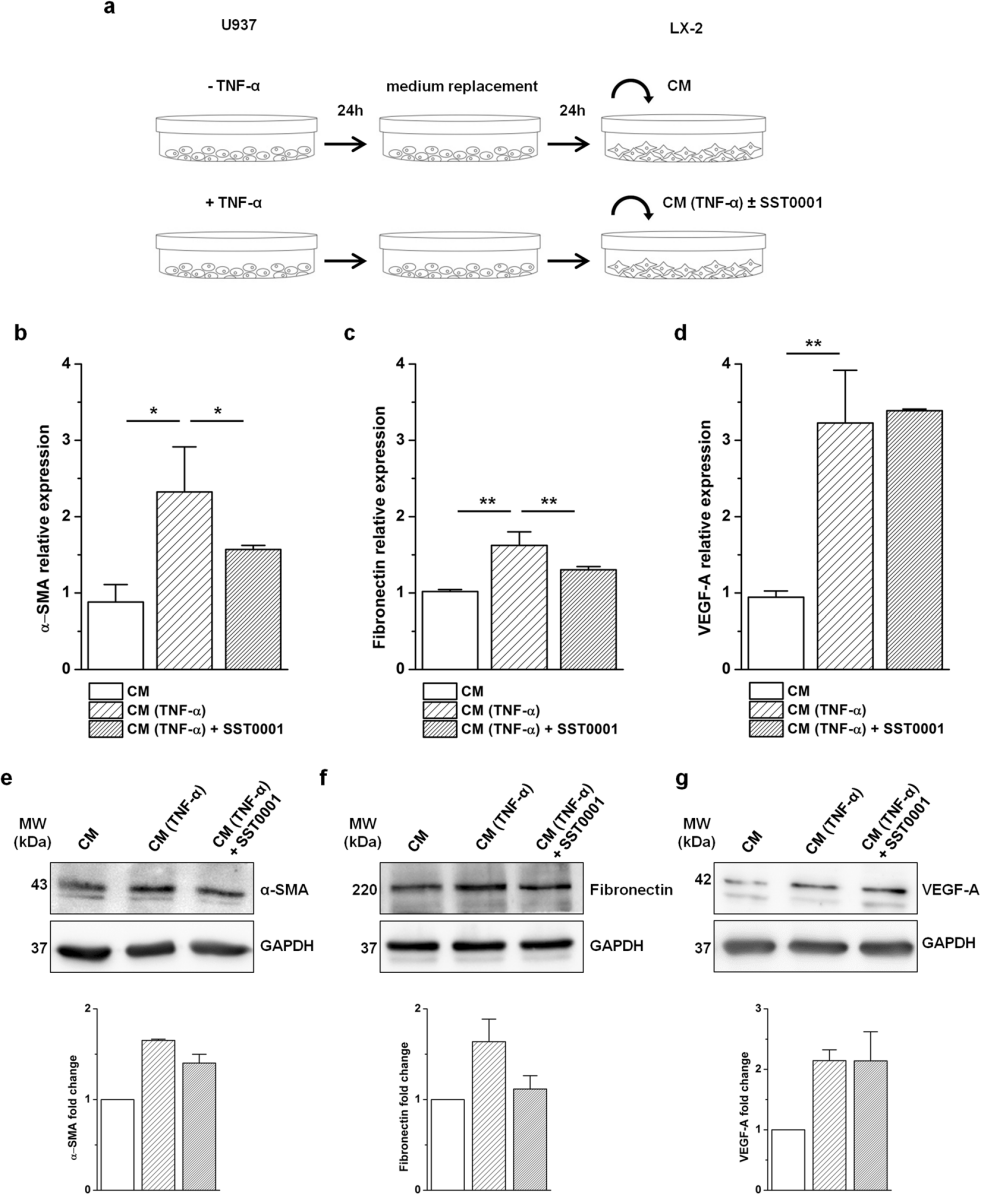
HPSE regulation of HSCs activation. (**a)** Experimental protocol of conditioned medium experiment. U937 macrophages were incubated without or with TNF-α for 24 h after which they were maintained 24 h in medium without serum. Conditioned media from untreated (CM) and pre-treated (CM (TNF-α)) U937 were collected and transferred to serum starved LX-2 cells. Conditioned medium from pre-treated U937 was added without or with SST0001. The expression of α-SMA, fibronectin and VEGF-A was detected in LX-2 treated with CM and CM (TNF-α) ± SST0001 by real-time RT-PCR **(b,c,d)** and Western blot **(e,f,g)**. Error bars represent s.e.m., *n* = 3, **p < 0.01, *p < 0.05. Original blots are presented in Supplementary Fig. S9.

To understand whether macrophage-derived HPSE could be a regulator of HSCs activation, LX-2 cells were grown in the conditioned medium of U937 macrophages pre-treated with or without TNF-α to induce HPSE expression. To verify the influence of HPSE on cell activation, U937 conditioned medium was added in the presence or absence of the heparin-derived HPSE inhibitor SST0001 (Fig. 5a). The conditioned medium of TNF-α-activated macrophages induced a significant up-regulation of α-SMA and fibronectin mRNA levels as compared to treatment with the conditioned medium of untreated U937. However, when LX-2 cells were treated with the conditioned medium of TNF-α-activated macrophages in combination with SST0001, the increases of both α-SMA and fibronectin mRNA were significantly diminished (Fig. 5b,c). These transcriptional data were also confirmed at protein level by Western blot analyses (Fig. 5e,f). In contrast to α-SMA and fibronectin, no significant difference was observed in the expression of the HSCs marker collagen 1(α1), both in the presence or absence of SST0001 (Supplementary Fig. S4). Among soluble mediators, VEGF is one of the major growth factors expressed and secreted by activated HSCs^30^. Since HPSE is known to regulate VEGF expression^31,32^, we investigated if HPSE could also modulate VEGF transcription in HSCs. The expression of VEGF increased in LX-2 stimulated with the conditioned medium of TNF-α activated U937 *vs*. cells stimulated with the conditioned medium of untreated U937. The addition of SST0001, however, did not affect VEGF expression, as observed both at mRNA and protein levels (Fig. 5d, 5g). These results imply that HPSE may modulate HSCs activation and highlight a new mechanism by which inflammatory macrophages control HSCs activity.

### HPSE plasma activity inversely correlated with human liver stiffness

**Figure 6:**
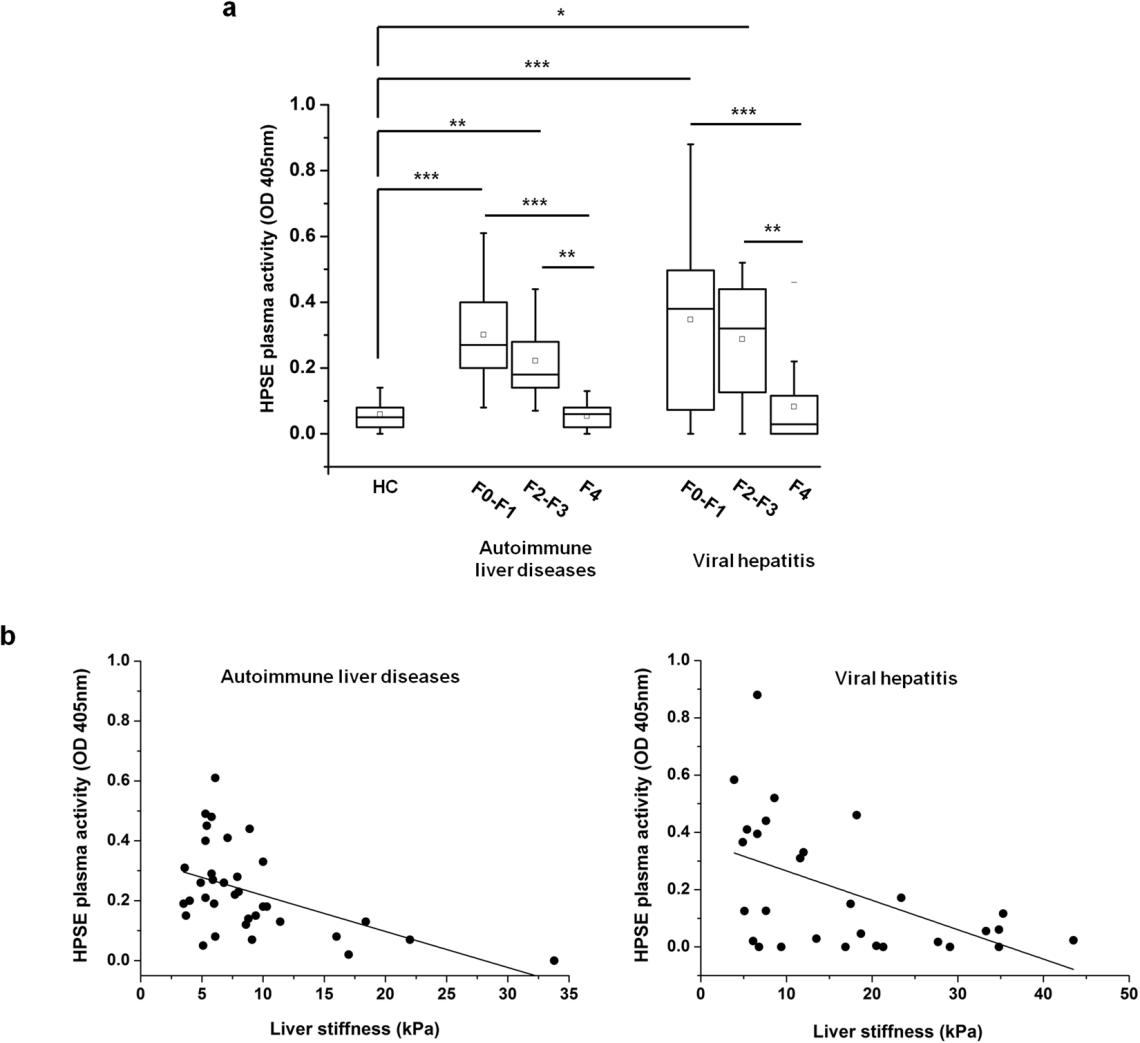
HPSE plasma activity in patients with chronic liver diseases and its correlation with liver stiffness. **(a)** Box-plot of HPSE activity in the plasma of healthy controls (HC) (*n = *14) and patients with autoimmune liver diseases (*n* = 35) and viral hepatitis (*n* = 30). Patients were staged from F0 to F4. Squares represent mean values. ***p < 0.001, **p < 0.01, *P < 0.05. **(b)** Scatter-plots of HPSE activity quantified in the plasma of patients with autoimmune liver diseases (left) and viral hepatitis (right) and correlated to liver stiffness. Trend lines were reported.

To further investigate the role of HPSE in the development of liver fibrosis, we studied and correlated HPSE activity measured in the plasma of patients with chronic liver disease and the stage of the disease itself, assessed by transient elastography. HPSE plasma activity was higher in patients with autoimmune liver disease at F0-F1 and F2-F3 stages of fibrosis with respect to healthy control group and these increases were statistically significant. However, HPSE plasma activity did not increase in patients with F4 fibrosis stage compared to controls. In patients with chronic viral hepatitis, HPSE plasma activity was elevated in F0-F1 and F2-F3 fibrotic compared to healthy controls and these increases were statistically significant. As observed in patients with autoimmune liver diseases, F4 fibrotic patients affected by hepatitis C (HCV) and hepatitis B (HBV) also had basal plasma HPSE activity (Fig. 6a). In addition, an inverse correlation of HPSE activity with organ stiffness was found, both in autoimmune liver diseases (R = 0.23, p = 0.002) and viral hepatitis (R = 0.24, p = 0.004) (Fig. 6b).

## Discussion

As an ECM remodeling enzyme with the ability to cleave HS chains, HPSE is determinant in those processes that require cell movement and growth factors bioavailability (embryogenesis, angiogenesis and tumor progression)^8,9^. Interest in HPSE in acute and chronic inflammatory disease and cancer-related inflammation has increased in the last decade and has led to the discovery of novel pro-inflammatory and pro-fibrogenic roles for this enzyme^6,33,34^. Interesting data have emerged from chronic colitis and diabetic nephropathy mouse models, both supporting the hypothesis that a chronic inflammatory circuit, connecting injured epithelial cells and inflammatory macrophages, is mediated by HPSE^17,35^. Taken together, these investigations have highlighted the role of HPSE in sustaining inflammation and fibrosis, which we hypothesized could be translated to the liver context. Specifically, we questioned whether HPSE could be up-regulated in or by injured liver and which could be the implications for liver fibrogenesis. With a set of *in vivo*, *in vitro* and *ex vivo* experiments, we provided evidence that HPSE was crucially involved in the establishment of chronic liver injury. Indeed, our data showed that HPSE underwent up-regulation both at gene and protein level in liver tissue of early chronic CCl_4_-treated mice but progressively decreased in the course of liver injury. Interestingly, HPSE levels did not increase concomitantly with the development of fibrosis. This trend in HPSE expression suggests that this protein could be involved in the first stage of liver disease onset. HPSE expression was also restricted in terms of spatial distribution, being detected at the necrotic and inflamed centrilobular zones and where it localized with macrophages. Thus, at variance from inflammatory bowel diseases and diabetic nephropathy where HPSE increase is preferentially sustained by injured epithelial cells^11,17,35,36^, in our model HPSE was primarily expressed by inflammatory macrophages in the early phase of chronic liver injury. As far as the decline of HPSE in late fibrotic stages is concerned, we hypothesized a switching-off mechanism which could occur in macrophages according to their plastic phenotype. In addition, it would be interesting to analyze in depth whether other different inflammatory cell types, which participate in the initiation of liver fibrosis, could be a source of HPSE. In this view, neutrophils are an interesting cell population to be analyzed further, due to their critical but redundant role both in acute and chronic liver damage^37,38^. However, considering published data on the role of this population in also repairing chronic injured tissues and on macrophages-neutrophil mutual exclusive presence in the reparative phase^39,40^, it would be interesting to analyze in our model if the decrease in macrophage-derived HPSE is associated with a massive neutrophils presence.

A pro-fibrogenic role of HPSE has been demonstrated in kidney and, more recently, in intestine^23,41^. The observation of enhanced HPSE expression in the early stages of chronic liver disease led us to wonder what its role could be in the process of hepatic fibrogenesis and, consequently, the possible mechanism at the basis of this pathological event. In the initial phase of hepatic injury, Kupffer cells react to parenchymal damage by secreting inflammatory mediators (mainly TNF-α, IL-1β and TGF-β) that perpetuate inflammation and hepatocellular damage^42^. Here we proved that TNF-α, as well as TGF-β, but not IL-1β, increased HPSE production as well as its release by macrophages. Our data further support the ability of both TNF-α and TGF-β to induce HPSE expression and secretion that was already observed in endothelial cells^43^ and epithelial cells^17,22^. In a model of chronic colitis, Lerner *et al*. showed that macrophage-derived TNF-α induced HPSE expression in colon epithelium which, in turn, fostered macrophage activation^17^. In our model this is not verified. Indeed, two *in vivo* observations (first, TNF-α up-regulation, in line with HPSE expression, in injured liver only at 1 and 2 weeks of treatment and second, TNF-α strong immuno-colocalization with HPSE) together demonstrated a role for TNF-α in regulating HPSE expression in macrophages themselves and allow us to suppose that there is an autocrine loop between TNF-α and HPSE to maintain macrophage activation. Considering TGF-β modulation during the chronic liver injury, the role of TGF-β in HPSE modulation and in relation to TNF-α is under investigation.

In the complex cellular cross-talk that characterizes chronic liver injury, activated macrophages are a stimulus for HSCs activation at early steps, by the release of pro-fibrogenic growth factors^2^. Considering the ability of HPSE to regulate the availability of many HS-linked molecules^20^, the activity of macrophage-derived HPSE was also expected to modulate HSCs activation. Indeed, we provided evidence that HPSE participated in the up-regulation of fibrogenic markers α-SMA and fibronectin, but not collagen 1(α1), by activated LX-2 cells. These data further strengthened the pro-fibrogenic role of HPSE that we had already demonstrated in kidney fibrosis, in which, through the regulation of pro-fibrotic factor bioavailability, it modulates mesenchymal marker expression sustaining tubular transdifferentiation and fibrosis^21,22^. The fact that the inhibition of HPSE by means of SST0001 did not alter the expression of VEGF-A suggested to us that HPSE is not a direct regulator of stellate cells VEGF-A expression, but rather that other molecules released by activated macrophages in the microenvironment could participate in the pro-angiogenic behaviour of HSCs.

Considering the transient HPSE expression observed in the CCl_4_ mouse model of liver fibrosis, in the *ex vivo* part of this work HPSE activity was measured in the plasma of healthy subjects and patients with chronic liver disease at various stages of fibrosis, of viral or autoimmune etiology. HPSE activity was found elevated in the plasma of patients with mild, significant and severe fibrosis, whereas it decreased to basal levels in cirrhotic patients. Moreover, it is noteworthy that an inverse correlation between HPSE activity and liver stiffness was demonstrated in this cohort of patients. These findings strongly supported the *in vivo* data from the CCl_4_-animal model of fibrosis and provide further evidence of an early role played by HPSE in the establishment of an hepatic disease. The fact that these results were observed in patients with both autoimmune liver disease and viral hepatitis suggests that the pathological events that are at the basis of HPSE up-regulation are probably the same independently of the etiology of liver disease.

**Figure 7:**
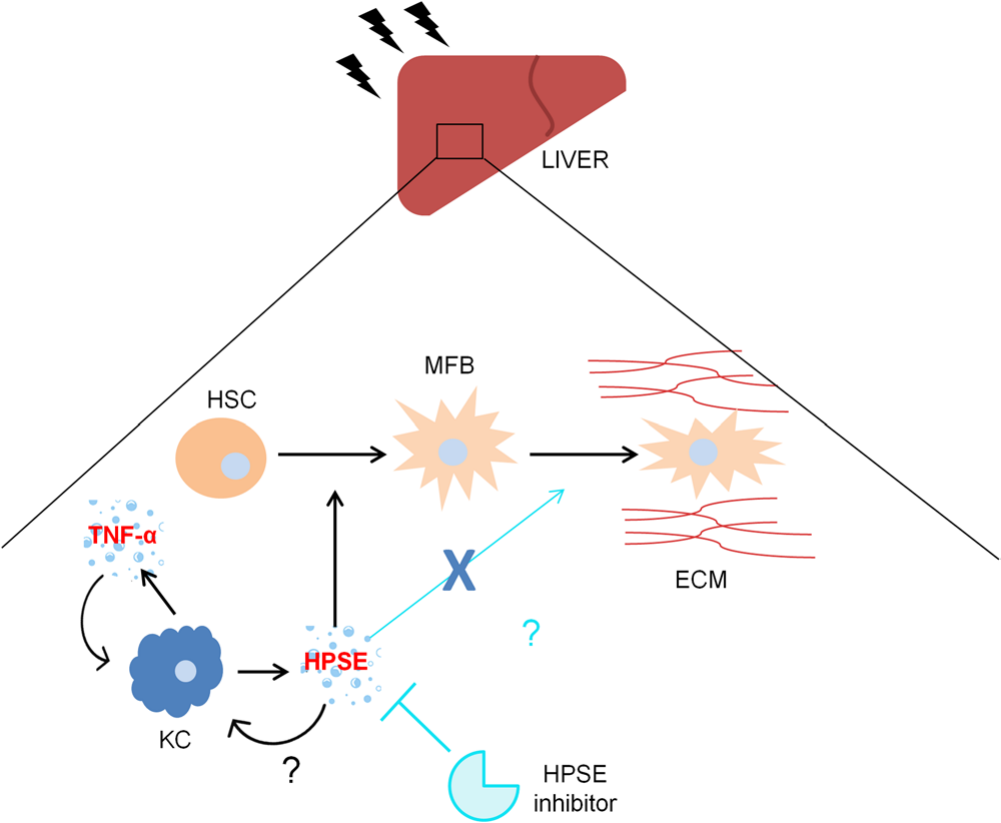
Mechanism of action of HPSE in liver fibrosis dynamics. In response to a chronic insult, hepatic Kupffer cells (KC) activate secreting inflammatory cytokines. Among them, TNF-α is the most critical in sustaining a positive loop of Kupffer cell activation. This leads to secretion of HPSE by macrophages which, in turn, regulates the activation of stellate cells (HSC) into myofibroblasts (MFB) to the extent of determining a dramatic alteration in extracellular matrix (ECM) composition. The inhibition of HPSE by a molecular drug could be a therapeutic strategy to counteract fibrosis onset.

Collectively, our supposed model of cascade of events can be summarized as follows. In response to injury, activated macrophages trigger inflammation by the secretion of inflammatory cytokines and chemokines. Among these, TNF-α, acting on macrophages themselves, induces the expression and secretion of HPSE. We hypothesize that macrophage-derived HPSE may sustain early steps of fibrogenesis through HSCs activation. It could also be plausible that HPSE enhances Kupffer cell activation since a role for HPSE in sensitizing macrophages to activation recently emerged in different inflammatory conditions^17,35^, involving both HS cleavage^44,45^ and non-enzymatic activity^46^. Further studies need to be performed to confirm the biological significance and the functional role of HPSE in chronic liver disease, especially in relation to macrophages as the main source of bioavailability. Furthermore, a deep analysis of fibrosis establishment in a mouse model of chronic liver injury in which an inhibitor for HPSE is used in a preventive setting is mandatory. The fact that HPSE seems to be involved in the initial steps of the disease acquires even more relevance if considering that the probability of liver fibrosis resolution decreases with advanced disease^1^. Bearing in mind the experimental evidences arising from our study, HPSE could be considered an early possible pharmacological target in the prevention of fibrosis establishment. Considering that several HPSE inhibitors are currently undergoing clinical trials and have demonstrated anti-tumor efficacy and few side effects, the results of this study strongly encourage a possible future use of these drugs in the treatment of liver fibrosis as well. A graphical abstract representing the hypothesised mechanism of action of HPSE in liver fibrosis and its role as a possible therapeutic target is shown in Fig. 7.

## Methods

### Animals

Twenty male Balb/cJ mice, 6 weeks old, were maintained in a pathogen-free and temperature-controlled environment at 12 h light/dark cycle and were fed with a standard rodent diet and water *a* *d libitum*. Animal studies followed the guidelines of the National Institutes of Health (CEASA protocol number 108288/2013, approved by the Ethics Committee of the University of Padova, Italy).

### Experimental model of liver fibrosis

Mice were randomly divided into CCl_4_-treated (*n* = 12) and control (*n = *8) groups. Animals were intraperitoneally injected with a mixture 1:7 of CCl_4_ (Sigma-Aldrich, Milan, Italy) in olive oil, at a dose of 1,2 μL/g body weight twice a week and sacrificed after 1, 2, 8 and 12 weeks from the beginning of the treatment. Control mice received intraperitoneal injections of oil. Animals (2 controls and 3 treated mice at each time point) were sacrificed and livers were harvested 72 h after the last injection of CCl_4_.

### Histopathology

Mouse liver tissue was formalin-fixed and paraffin-embedded. Five μm thick sections were stained with Hematoxylin-Eosin (H&E) and Azan-Mallory according to standard protocols.

### Cell culture

U937 human monocyte and LX-2 human HSCs cell lines were cultured in RPMI-1640 medium (Euroclone, Milan, Italy) supplemented with 10% fetal bovine serum, 2 mM L-glutamine, 100 U penicillin and 100 μg/mL streptomycin. Prior to each experiment, U937 suspension monocytes were differentiated in adherent macrophages by treatment with 20 ng/mL phorbol myristate acetate (PMA, Sigma-Aldrich, Saint Louis, Missouri, USA) for 48 h. U937 macrophages were serum starved and treated with 10 ng/mL TNF-α (T6674, Sigma-Aldrich) and 10 ng/mL active IL-1β (200-01B, Peprotech, London, UK). TGF-β (#8915, Cell Signaling Technology, Leiden, The Netherlands) was used at 20 ng/mL. For conditioned media experiments, U937 macrophages were seeded in 100 mm dishes and serum starved for 24 h. Cells were treated with or without TNF-α (10 ng/mL) for 24 h after which medium was replaced with fresh medium without serum for another 24 h. Conditioned media were collected, filtered (0.22 μm) and used to treat LX-2 cells in the presence or absence of the HPSE inhibitor SST0001 (500 μg/mL), a non-anticoagulant 100% N-acetylated, 25% glycol-split heparin (Leadiant Biosciences Ltd).

### Western blot

Snap-frozen liver samples were homogenized in RIPA buffer (150 mM NaCl, 1% Triton X-100, 0.5% sodium deoxycholate, 0.1% SDS, 50 mM Tris-HCl, pH 8) supplemented with Protease Inhibitor Cocktail (Sigma-Aldrich). Cells were homogenized in lysis buffer containing 1% Triton, 150 mM NaCl, 20 mM Tris-HCl, pH 7.5. Protein concentration was determined using a bicinchoninic acid assay kit (Thermo Scientific Pierce BCA Protein Assay Kit, Thermo Scientific, Rockford, USA). Equal amounts of protein lysate or equal volumes of conditioned media were mixed with Laemmli loading buffer and heated at 100 °C for 10 min. Proteins were subjected to SDS-PAGE on 10% acrylamide gel and electro-transferred to a nitrocellulose membrane (Sartorius AG, Goettingen, Germany). Membranes were blocked with 5% non-fat milk in Tris buffered saline with 0,1% Tween-20 (0,1% TBST) and incubated with primary antibody overnight at 4 °C. After washing with TBST, membranes were incubated with horseradish peroxidase (HRP)-conjugated secondary antibody for 90 min at room temperature (RT). Detection was performed using a chemiluminescence substrate with Alliance system (UVItec, Cambridge, UK). The following primary antibodies were used: anti-human HPSE (ANT-193, ProSpec, Ness-Ziona, Israel), anti-mouse HPSE (sc-25826, Santa Cruz Biotechnology, Santa Cruz, CA, USA), anti-VEGF (sc-152, Santa Cruz Biotechnology), anti-α-SMA (A5228, Sigma-Aldrich), anti-fibronectin (sc-9068, Santa Cruz Biotechnology) and anti-GAPDH (sc-25778, Santa Cruz Biotechnology). The following secondary antibodies were used: goat anti-rabbit IgG-HRP and goat anti-mouse IgG-HRP (sc-2004 and sc-2005 respectively, Santa Cruz Biotechnology).

### Immunohistochemistry

After deparaffinization, antigen unmasking was performed by heating slices in sodium citrate buffer (10 mM sodium citrate, 0.05% Tween-20, pH 6), in a microwave oven twice for 5 min. Slices were left 30 min at RT and washed 3 times for 5 min in dH_2_O. Inactivation of endogenous peroxidase was performed by using 3% H_2_O_2_ for 10 min. After washing with 0.1% TBST, sections were saturated with 5% goat serum in 0.1% TBST for 90 min at RT and incubated with primary antibody anti-HPSE (sc-25826, Santa Cruz) diluted 1:300 in antibody buffer (2.5% goat serum in 0.1% TBST) overnight at 4 °C. Then, slices were rinsed three times with 0.1% TBST for 5 min and incubated with a biotinylated goat anti-rabbit IgG secondary antibody (BA-1000, Vector laboratories) diluted 1:200 in antibody buffer for 30 min at RT. After washing with 0.1% TBST, sections were incubated with Streptavidin-HRP conjugate (Sigma-Aldrich) diluted 1:100 for 30 min. For signal detection, enzymatic reaction was developed using a substrate diaminobenzidine (DAB) solution (Sigma-Aldrich) for 1 min. Nuclei were counterstained with hematoxylin. Bright-field images were acquired using a Leica DMR microscope.

### Immunofluorescence

After deparaffinization and antigen unmasking, liver sections were permeabilized with 0.2% Triton X-100 in PBS for 10 min at RT and blocked in PBS supplemented with 1% BSA and 0.1% Triton X-100 for 1 h at RT. For double immunofluorescence, slices were incubated overnight at 4 °C with anti-HPSE antibody (sc-25826, Santa Cruz) in combination with anti-F4/80 (ab186073, Abcam, Cambridge, UK) or anti-TNF-α (sc-1350, Santa Cruz Biotechnology) antibodies. A5228 antibody (Sigma-Aldrich) was used for α-SMA detection. Antibodies were prepared in PBS supplemented with 1% BSA and 0.3% Triton X-100. Goat anti-rabbit Alexa Fluor 488, goat anti-chicken Alexa Fluor 546, donkey anti-goat Alexa Fluor 633 (A-11034, A-11040 and A-21082 respectively, Thermo Scientific) and goat anti-mouse Alexa Fluor 594 (ab150116, Abcam) were used as secondary antibodies. Nuclei were counterstained with Hoechst for 20 min at RT. Images were acquired using a confocal microscope Leica TCS SP5.

### Real-time reverse transcriptase-PCR

Total RNA from liver tissue and cell cultures was extracted using TRIzol reagent (Sigma-Aldrich), according to the manufacturer’s instructions. Five hundred ng of total RNA were reverse-transcribed using SuperScript II Reverse Transcriptase (Invitrogen, Carlsbad, USA) according to the manufacturer’s instructions. Quantitative real-time reverse transcriptase-polymerase chain reaction (real-time RT-PCR) was performed on the ABI 7900HT Fast system (Applied Biosystem, Milan, Italy) using a SYBR-Green Master Mix (BIOLINE, London, UK). Each reaction mix was processed using the following PCR conditions: polymerase activation at 95 °C for 2 min followed by 40 cycles of 95 °C for 5 sec (denaturation) and 60–62 °C for 20 sec (combined annealing/extension). Gene expression was normalized to GAPDH and quantified by the ΔΔC_T_ method. All reactions were performed in duplicates for each sample. Primer sequences are reported in Table 1.

**Table 1:** List of primers used for real time RT-PCR.

Gene	Primer sequence (5′–3′)	Product length (bp)
Mouse *GAPDH*	Forward: GGCAAATTCAACGGCACAGT Reverse: GTCTCGCTCCTGGAAGATGG	84
Mouse *HPSE*	Forward: GTTCCTGTCCATCACCATCGA Reverse: CTTGGAGAGCCCAGGAAGGT	72
Mouse *TNF-α*	Forward: CATCTTCTCAAAATTCGAGTGACAA Reverse: TGGGAGTAGACAAGGTACAACCC	175
Mouse *TGF-β*	Forward: GTGTGGAGCAACATGTGGAACTCTA Reverse: CGCTGAATCGAAAGCCCTGTA	174
Human *GAPDH*	Forward: ACACCCACTCCTCCACCTTT Reverse: TCCACCACCCTGTTGCTGTA	112
Human *HPSE*	Forward: CCCTTGCTATCCGACACCTT Reverse: CACCACTTCTATTCCCATTCG	84
Human *ACTA2*	Forward: TACTACTGCTGAGCGTGAGA Reverse: CATCAGGCAACTCGTAACTC	131
Human *VEGFA*	Forward: AGACGTGTAAATGTTCCTGCAAAA Reverse: TGCAAGTACGTTCGTTTAACTCAAG	78
Human *FN1*	Forward: GTGTGTTGGGAATGGTCGTG Reverse: GACGCTTGTGGAATGTGTCG	113
Human *COL1A1*	Forward: CAGACTGGCAACCTCAAGAAG Reverse: CGGTGTGACTCGTGCAGCCAT	121

### Human subjects

Seventy-nine subjects were consecutively enrolled in the study. Fourteen healthy volunteers (controls) and 65 patients with different chronic liver diseases were included. Patients were diagnosed for HBV (*n* = 7), HCV (*n* = 23), primary biliary cholangitis (PBC, *n* = 17), primary sclerosing cholangitis (PSC, *n* = 9), autoimmune hepatitis (AIH, *n* = 6), PBC/AIH overlap (*n* = 2), PSC/AIH overlap (*n* = 1), according to the European Association for the Study of the Liver (EASL) guidelines. Patients with HCV and HBV were categorized in viral hepatitis subgroup. Patients with PBC, PSC, AIH and overlap syndromes were categorized in autoimmune disease subgroup. The stage of liver fibrosis was assessed by transient elastography (Fibroscan, Echosens, Paris, France). The study was conducted according to the Declaration of Helsinki. All patients gave written consent for data collection and sample analysis. The study was approved by the Padua University-Hospital ethical committee (protocol number: 3212/A0/14).

### Plasma collection

A total of 3 mL of peripheral blood were collected from patients and healthy subjects. Blood was processed by centrifugation at 1500 × g for 15 min. Plasma were collected, aliquoted and stored at −20 °C until use.

### HPSE activity assay

HPSE plasma activity was measured using an ELISA-modified assay performed on plates covered with Matrigel (BD Bioscience, Erembodegem, Belgium), as previously described^47^.

### Statistical analysis

Data were reported as means ± standard error of the mean (s.e.m.). For comparison between two distributions, the two-tailed t-test was used. For multiple comparisons, the one-way analysis of variance (ANOVA) was used. Statistical analyses on real time RT-PCR data were performed using the Relative Expression Software Tool (REST). p-values < 0.05 were considered statistically significant.

### Data availability

All data generated or analysed during this study are included in this published article (and its Supplementary Information files).

## Electronic supplementary material

Supplementary graphs and figures

## References

[1] Pellicoro A, Ramachandran P, Iredale JP, Fallowfield JA (2014). Liver fibrosis and repair: immune regulation of wound healing in a solid organ. Nature reviews. Immunology.

[2] Elpek GO (2014). Cellular and molecular mechanisms in the pathogenesis of liver fibrosis: An update. World journal of gastroenterology.

[3] Puche JE, Saiman Y, Friedman SL (2013). Hepatic stellate cells and liver fibrosis. Comprehensive Physiology.

[4] Dixon LJ, Barnes M, Tang H, Pritchard MT, Nagy LE (2013). Kupffer cells in the liver. Comprehensive Physiology.

[5] Vreys V, David G (2007). Mammalian heparanase: what is the message?. Journal of cellular and molecular medicine.

[6] Secchi MF (2015). Recent data concerning heparanase: focus on fibrosis, inflammation and cancer. Biomolecular concepts.

[7] Nasser NJ (2008). Heparanase involvement in physiology and disease. Cellular and molecular life sciences: CMLS.

[8] Rivara S, Milazzo FM, Giannini G (2016). Heparanase: a rainbow pharmacological target associated to multiple pathologies including rare diseases. Future medicinal chemistry.

[9] Sanderson RD, Elkin M, Rapraeger AC, Ilan N, Vlodavsky I (2017). Heparanase regulation of cancer, autophagy and inflammation: new mechanisms and targets for therapy. The FEBS journal.

[10] Levidiotis V, Freeman C, Tikellis C, Cooper ME, Power DA (2004). Heparanase is involved in the pathogenesis of proteinuria as a result of glomerulonephritis. Journal of the American Society of Nephrology: JASN.

[11] van den Hoven MJ (2006). Increased expression of heparanase in overt diabetic nephropathy. Kidney international.

[12] Levidiotis V (2004). A synthetic heparanase inhibitor reduces proteinuria in passive Heymann nephritis. Journal of the American Society of Nephrology: JASN.

[13] Rabelink, T. J. *et al*. Heparanase: roles in cell survival, extracellular matrix remodelling and the development of kidney disease. *Nature reviews. Nephrology*, doi:10.1038/nrneph.2017.6 (2017). 10.1038/nrneph.2017.6 28163306

[14] Nadir Y, Brenner B (2014). Heparanase multiple effects in cancer. Thrombosis research.

[15] Masola V, Secchi MF, Gambaro G, Onisto M (2014). Heparanase as a target in cancer therapy. Current cancer drug targets.

[16] Vlodavsky I (2016). Heparanase: From basic research to therapeutic applications in cancer and inflammation. Drug resistance updates: reviews and commentaries in antimicrobial and anticancer chemotherapy.

[17] Lerner I (2011). Heparanase powers a chronic inflammatory circuit that promotes colitis-associated tumorigenesis in mice. The Journal of clinical investigation.

[18] Shimshoni E, Yablecovitch D, Baram L, Dotan I, Sagi I (2015). ECM remodelling in IBD: innocent bystander or partner in crime? The emerging role of extracellular molecular events in sustaining intestinal inflammation. Gut.

[19] Turnbull J, Powell A, Guimond S (2001). Heparan sulfate: decoding a dynamic multifunctional cell regulator. Trends in cell biology.

[20] Manon-Jensen T, Itoh Y, Couchman JR (2010). Proteoglycans in health and disease: the multiple roles of syndecan shedding. The FEBS journal.

[21] Masola V (2012). Heparanase and syndecan-1 interplay orchestrates fibroblast growth factor-2-induced epithelial-mesenchymal transition in renal tubular cells. The Journal of biological chemistry.

[22] Masola V (2014). Heparanase is a key player in renal fibrosis by regulating TGF-beta expression and activity. Biochimica et biophysica acta.

[23] Gil N (2012). Heparanase is essential for the development of diabetic nephropathy in mice. Diabetes.

[24] Xiao Y, Kleeff J, Shi X, Buchler MW, Friess H (2003). Heparanase expression in hepatocellular carcinoma and the cirrhotic liver. Hepatology research: the official journal of the Japan Society of Hepatology.

[25] El-Assal ON, Yamanoi A, Ono T, Kohno H, Nagasue N (2001). The clinicopathological significance of heparanase and basic fibroblast growth factor expressions in hepatocellular carcinoma. Clinical cancer research: an official journal of the American Association for Cancer Research.

[26] Liu CJ (2014). Adjuvant heparanase inhibitor PI-88 therapy for hepatocellular carcinoma recurrence. World journal of gastroenterology.

[27] Ikeguchi, M., Hirooka, Y. & Kaibara, N. Heparanase gene expression and its correlation with spontaneous apoptosis in hepatocytes of cirrhotic liver and carcinoma. *European journal of cancer (Oxford, England: 1990)* **39**, 86–90 (2003). 10.1016/s0959-8049(02)00558-0 12504663

[28] Goldshmidt O (2004). Heparanase expression during normal liver development and following partial hepatectomy. The Journal of pathology.

[29] Ohayon O (2008). Halofuginone upregulates the expression of heparanase in thioacetamide-induced liver fibrosis in rats. Laboratory investigation; a journal of technical methods and pathology.

[30] Zhao Y (2012). Hepatic stellate cells produce vascular endothelial growth factor via phospho-p44/42 mitogen-activated protein kinase/cyclooxygenase-2 pathway. Molecular and cellular biochemistry.

[31] Zetser A (2006). Heparanase induces vascular endothelial growth factor expression: correlation with p38 phosphorylation levels and Src activation. Cancer research.

[32] Hu J (2012). *Heparanase and vascular endothelial growth fact*or expression is increased in hypoxia-induced retinal neovascularization. Investigative ophthalmology & visual science.

[33] Meirovitz A (2013). Heparanase in inflammation and inflammation-associated cancer. The FEBS journal.

[34] Masola V, Zaza G, Onisto M, Lupo A, Gambaro G (2015). Impact of heparanase on renal fibrosis. Journal of translational medicine.

[35] Goldberg R (2014). Role of heparanase-driven inflammatory cascade in pathogenesis of diabetic nephropathy. Diabetes.

[36] Waterman M (2007). Heparanase upregulation by colonic epithelium in inflammatory bowel disease. Modern pathology: an official journal of the United States and Canadian Academy of Pathology, Inc.

[37] Tan, Z. *et al*. IL-17A plays a critical role in the pathogenesis of liver fibrosis through hepatic stellate cell activation. *Journal of immunology (Baltimore, M. d.: 1950*) **19**1, 1835–1844, doi:10.4049/jimmunol.1203013 (2013). 10.4049/jimmunol.1203013 23842754

[38] Moles A (2014). A TLR2/S100A9/CXCL-2 signaling network is necessary for neutrophil recruitment in acute and chronic liver injury in the mouse. Journal of hepatology.

[39] Harty MW (2008). Hepatic macrophages promote the neutrophil-dependent resolution of fibrosis in repairing cholestatic rat livers. Surgery.

[40] Harty MW (2010). Neutrophil depletion blocks early collagen degradation in repairing cholestatic rat livers. The American journal of pathology.

[41] Davids, J. S., Carothers, A. M., Damas, B. C. & Bertagnolli, M. M. Chronic cyclooxygenase-2 inhibition promotes myofibroblast-associated intestinal fibrosis. *Cancer prevention research (Philadelphia, Pa.)* **3**, 348–358, doi:10.1158/1940-6207.capr-09-0146 (2010). 10.1158/1940-6207.CAPR-09-0146 PMC2833233 20179298

[42] Tacke F, Zimmermann HW (2014). Macrophage heterogeneity in liver injury and fibrosis. Journal of hepatology.

[43] Chen G (2004). Inflammatory cytokines and fatty acids regulate endothelial cell heparanase expression. Biochemistry.

[44] Goodall KJ, Poon IK, Phipps S, Hulett MD (2014). Soluble heparan sulfate fragments generated by heparanase trigger the release of pro-inflammatory cytokines through TLR-4. PloS one.

[45] Blich M (2013). Macrophage activation by heparanase is mediated by TLR-2 and TLR-4 and associates with plaque progression. Arteriosclerosis, thrombosis, and vascular biology.

[46] Gutter-Kapon L (2016). Heparanase is required for activation and function of macrophages. Proceedings of the National Academy of Sciences of the United States of America.

[47] Zaza, G. *et al*. Dialysis-related transcriptomic profiling: the pivotal role of heparanase. *Experimental biology and medicine (Maywood, N. J.)* **239**, 52–64, doi:10.1177/1535370213506678 (2014). 10.1177/1535370213506678 24189015

