# Supplementary material for: Heparanase and macrophage interplay in the onset of liver fibrosis
Source: Sci Rep. 2017 Nov 2;7:14956. doi: 10.1038/s41598-017-14946-0 (PMC5668295; doi:10.1038/s41598-017-14946-0)
Supplement: Supplementary file 1 — Supplementary graphs and figures [file 41598_2017_14946_MOESM1_ESM.pdf]

Heparanase and macrophage interplay in the onset of liver fibrosis

Maria Francesca Secchi[1,2], Marika Crescenzi[2], Valentina Masola[1,3], Francesco Paolo Russo[2,*], Annarosa Floreani[2], Maurizio Onisto[1,*]

[1] University of Padova, Dept. Biomedical Sciences, 35121, Padova, Italy

[2] University of Padova, Dept. of Surgery, Oncology and Gastroenterology, 35124, Padova, Italy

[3] University of Verona, Dept. of Medicine, 37134, Verona, Italy

* maurizio.onisto@unipd.it, francescopaolo.russo@unipd.it

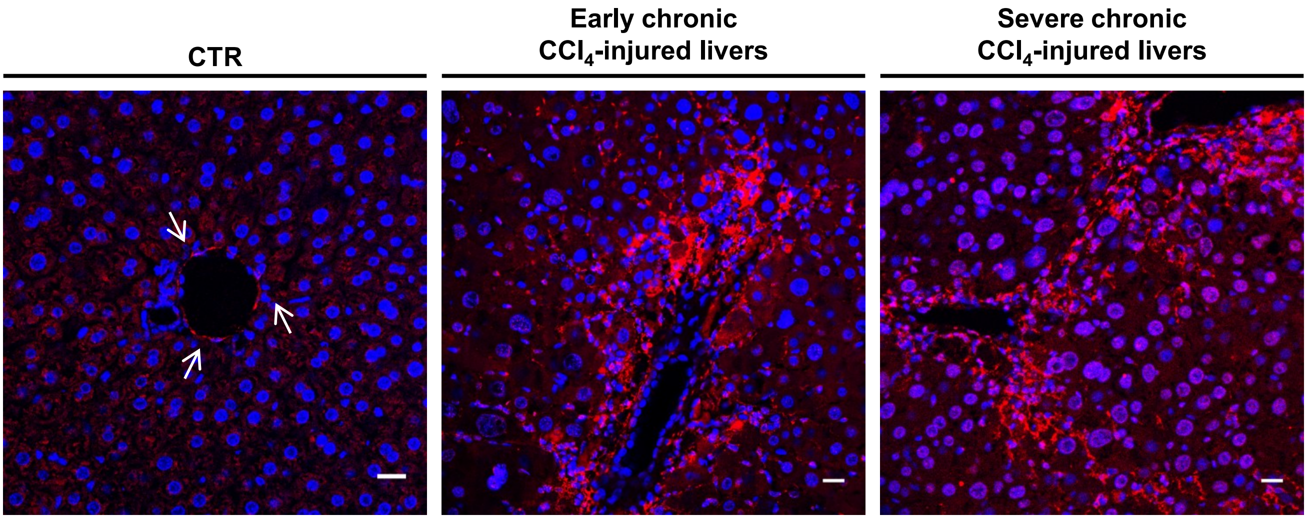

Figure S1. α-SMA immunofluorescence staining in CCl_4-injured liver tissues. Representative images of confocal immunofluorescence staining for α-SMA (red) on control livers, early chronic CCl_4-injured livers (1 week of CCl_4 administration) and severe chronic CCl_4-injured livers (8 weeks of CCl_4 administration). Cell nuclei were counterstained with Hoechst (blue). White arrows indicate positive vascular smooth muscle cells. Scale bar = 20μm.

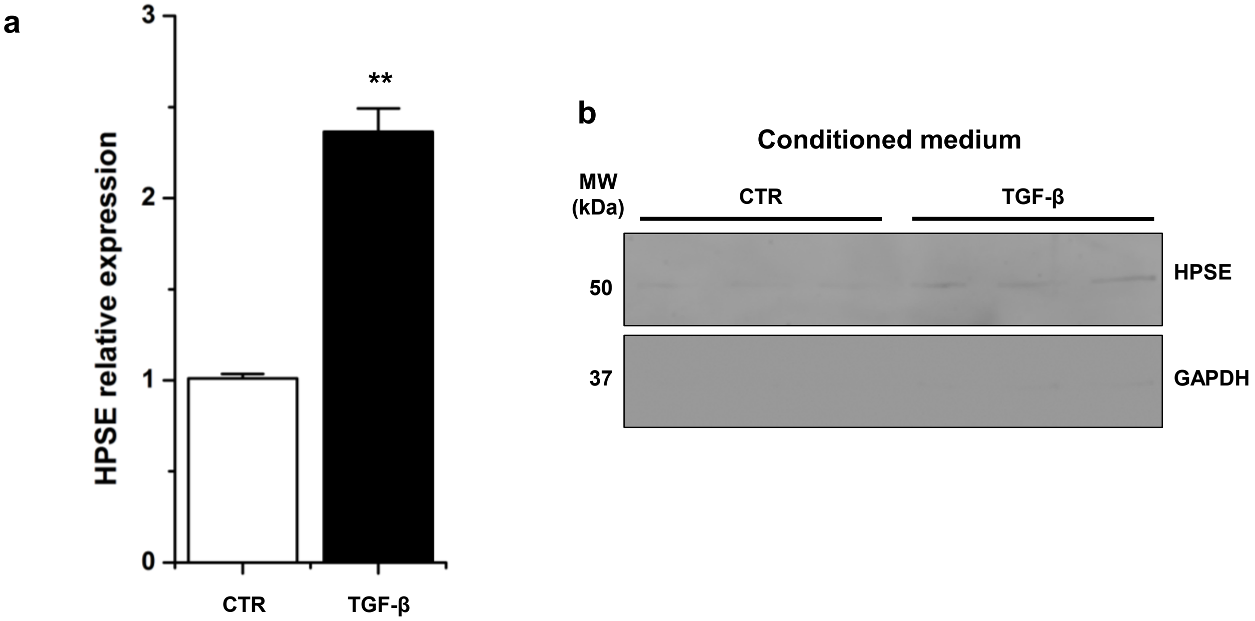

Figure S2. TGF-β regulation of HPSE expression in U937 macrophages. (a) U937 cells were treated with TGF-β for 24 h. The expression of HPSE was assessed by real-time RT-PCR and normalized to GAPDH. Error bars represent s.e.m., n = 3, ** $p < 0.01$. **(b)** Western blot analysis for HPSE on conditioned medium from control and TGF-β-treated U937 cells.

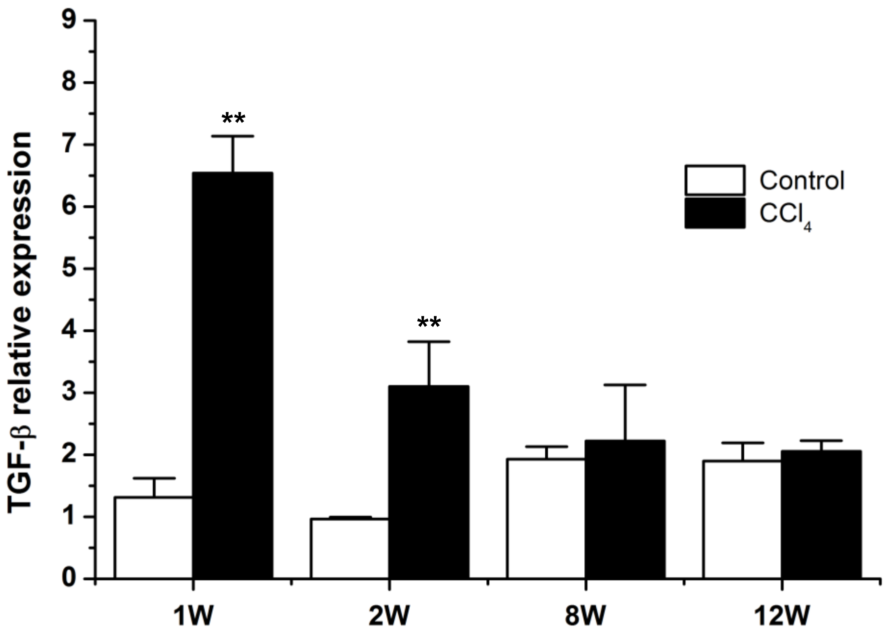

Figure S3. Expression of HPSE in mice with CCl_4-induced chronic liver disease. HPSE mRNA expression in livers from control and CCl_4-treated mice for the indicated time, measured by real-time RT-PCR analysis and normalized to GAPDH. Error bars represent s.e.m., n = 3, ** $p < 0.01$.

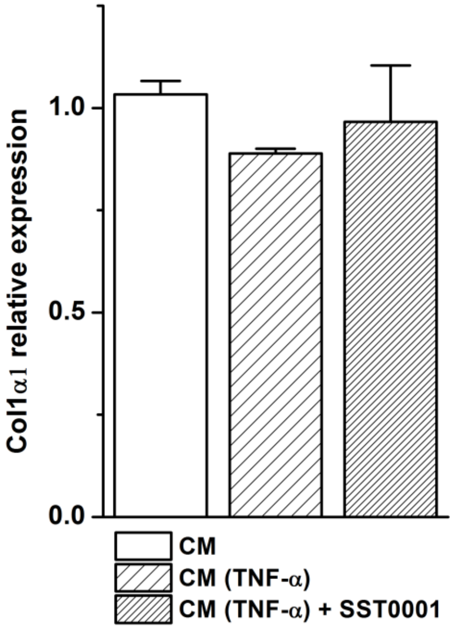

Figure S4. Expression of collagen 1(α1) in LX-2 cells treated with U937 conditioned medium (CM). The expression of Collagen 1(α1) was detected in LX-2 treated with CM and CM (TNF-α) ± SST0001 by real-time RT-PCR. Error bars represent s.e.m., n = 3.

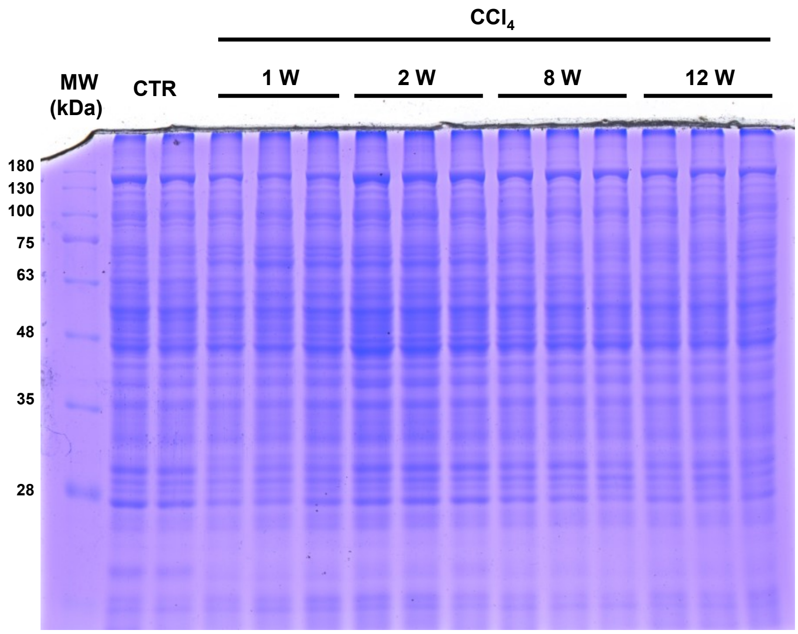

Figure S5. Coomassie blu staining as a loading control for western blot showed in Figure 1c.

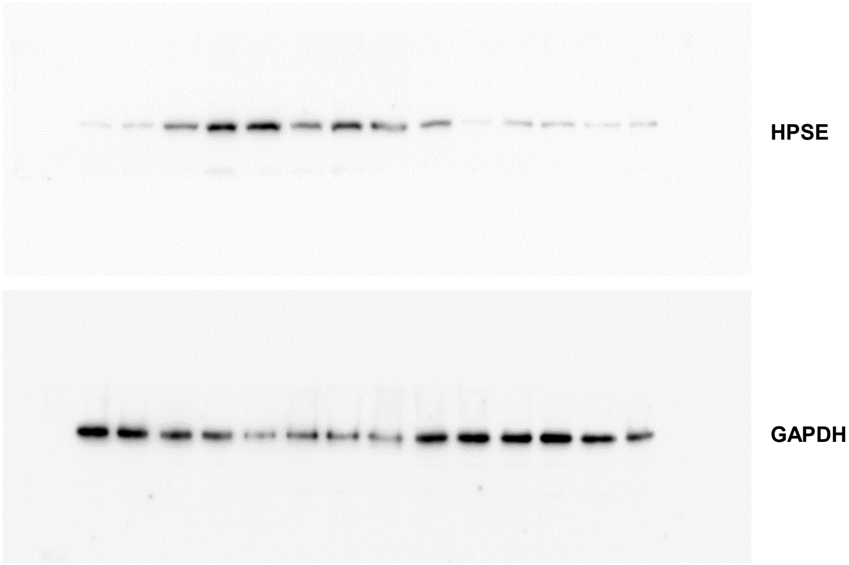

Figure S6. Full-length western blot shown in Fig. 1c.

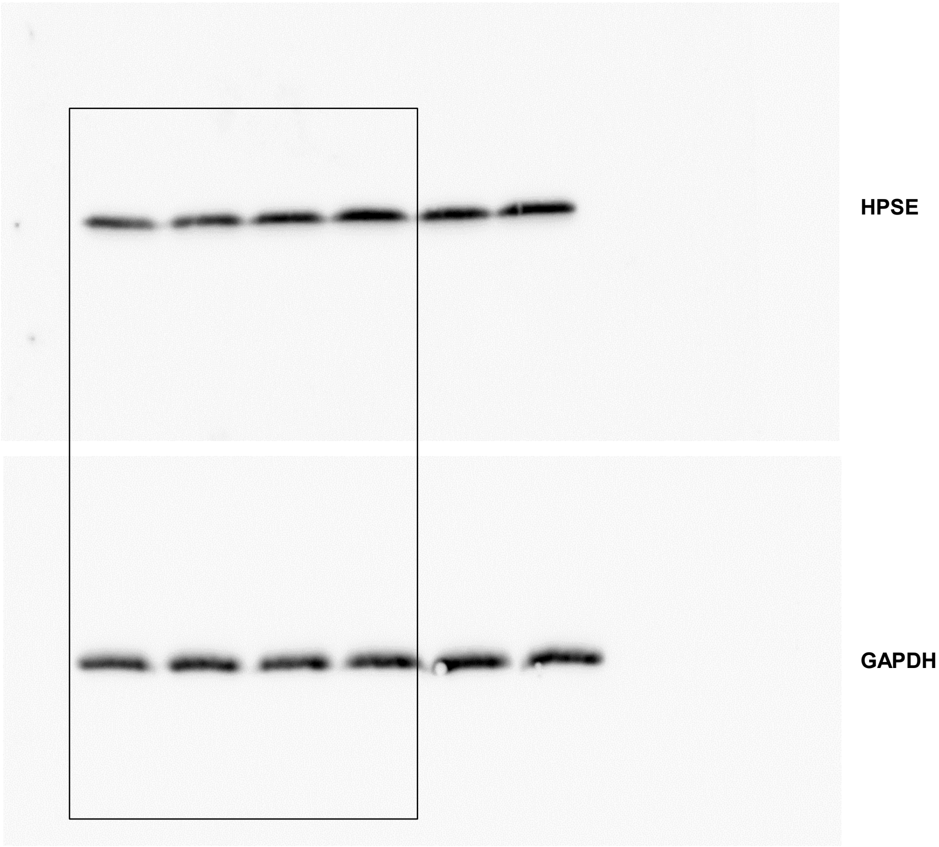

Figure S7. Full-length western blots shown in Fig. 3b.

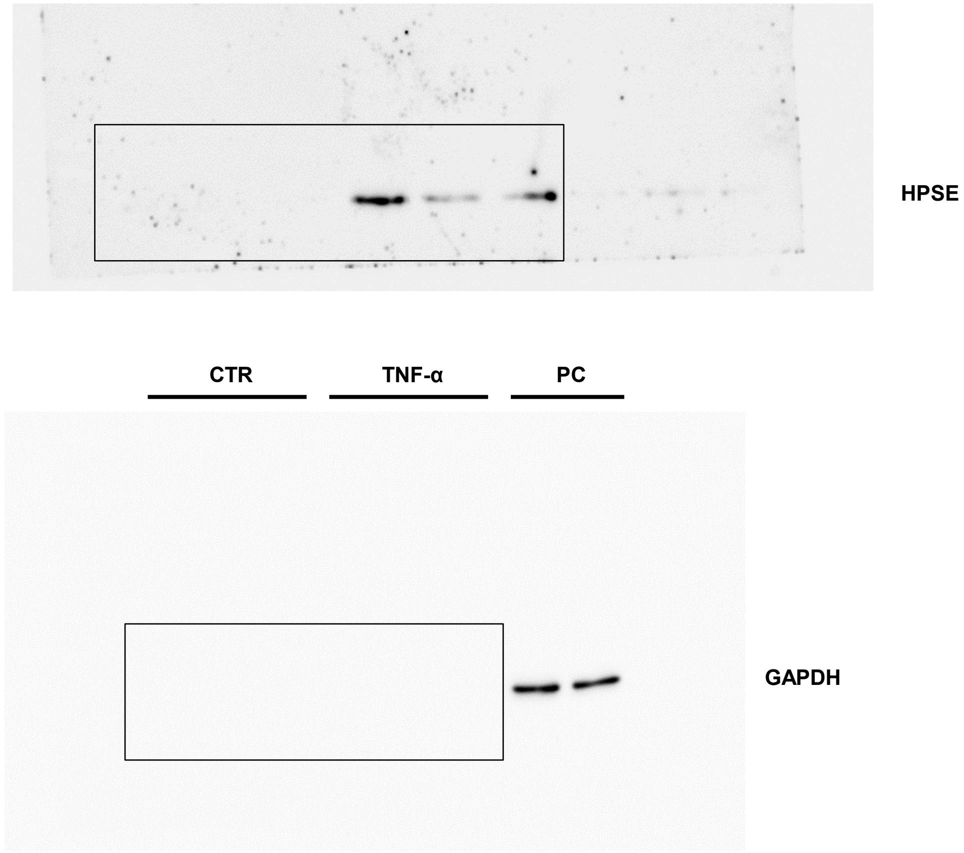

Figure S8. Full-length western blots shown in Fig. 3c. GAPDH positive controls (PC) are total protein extracts from U937 cells. HPSE and GAPDH were run on different gels.

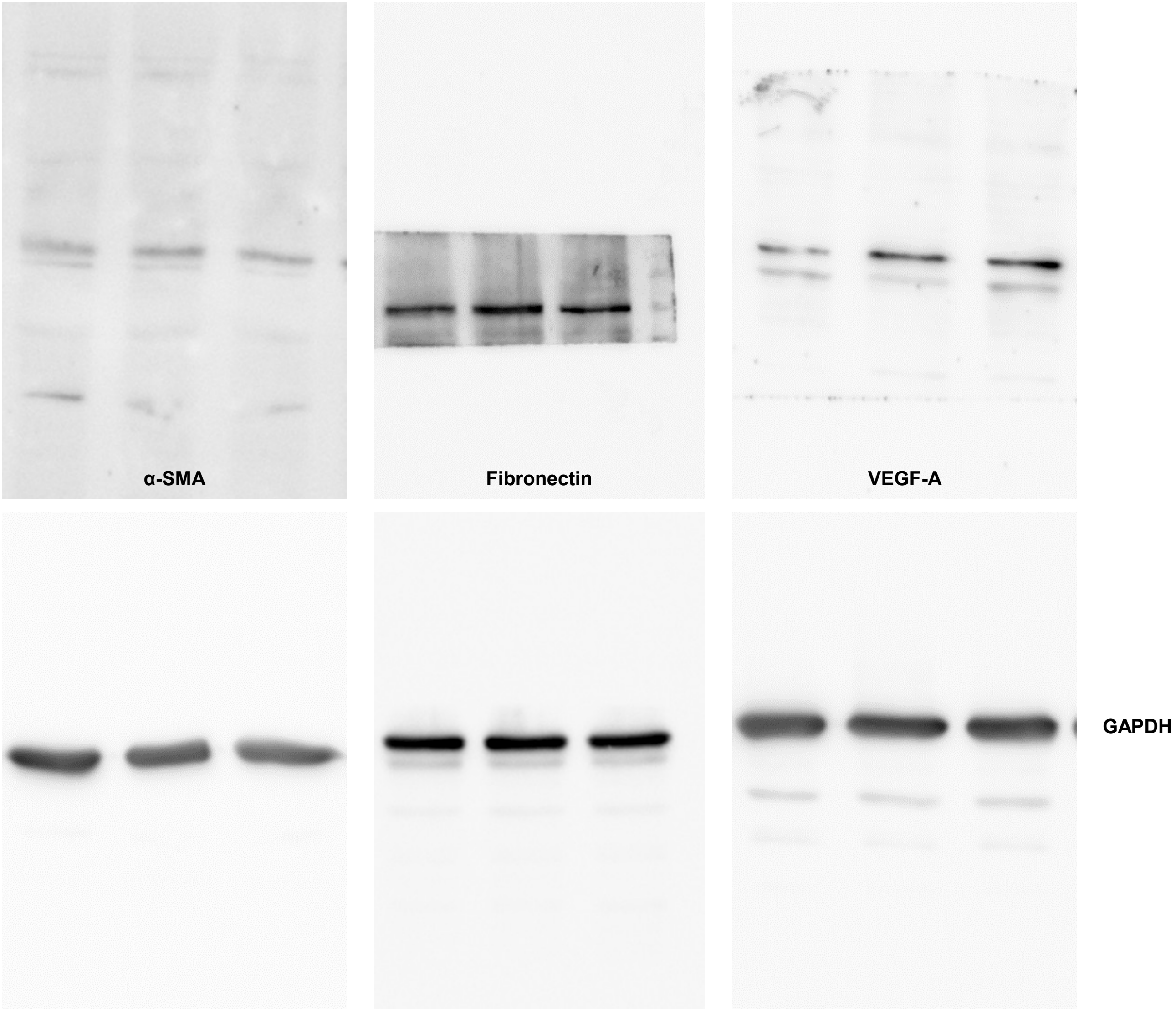

Figure S9. Original western blots shown in Fig. 5e,f,g.
